# Detection of trace concentrations of S-nitrosothiols by means of a capacitive sensor

**DOI:** 10.1371/journal.pone.0187149

**Published:** 2017-10-26

**Authors:** James M. Seckler, Nikki M. Meyer, Spencer T. Burton, James N. Bates, Benjamin Gaston, Stephen J. Lewis

**Affiliations:** 1 Department of Pediatrics, Case Western Reserve University, Cleveland, Ohio, United States of America; 2 Department of Anesthesia, University of Iowa, Iowa City, Iowa, United States of America; 3 Rainbow Babies and Children’s Hospital, Cleveland, Ohio, United States of America; Institute of Materials Science, GERMANY

## Abstract

Small molecule S-nitrosothiols are a class of endogenous chemicals in the body, which have been implicated in a variety of biological functions. However, the labile nature of NO and the limits of current detection assays have made studying these molecules difficult. Here we present a method for detecting trace concentrations of S-nitrosothiols in biological fluids. Capacitive sensors when coupled to a semiconducting material represent a method for detecting trace quantities of a chemical in complex solutions. We have taken advantage of the semiconducting and chemical properties of polydopamine to construct a capacitive sensor and associated method of use, which specifically senses S-nitrosothiols in complex biological solutions.

## Introduction

Small molecule S-nitrosothiols (SNOs) are generated by activating various forms of nitric oxide synthase and by interactions of nitric oxide (NO) with other metalloproteins [1]. The regulation and misregulation of these molecules has been shown to play a role in control of breathing, ventilation-perfusion matching, pulmonary hypertension, human airway smooth muscle tone, asthma, regulation of blood pressure, diabetes, and other metabolic diseases [1–4]. In all of these cases, the ability to measure and detect SNOs in biological samples is important in understanding their role in both normal function and disease states. However, SNOs normally exist at low nM levels in biology [5]. A fundamental problem in the field is that available assays are typically only sensitive to mid nM levels, and are therefore typically used near their limit of detection [6].

There are a variety of general biosensor methods that all consist of coupling a bioreceptor to a transducer [7]. Bioreceptors are the material used to recognize the biomolecule of interest, and they include antibodies [7,8,9], enzymes [7,10], molecularly imprinted polymers [7,11], aptamers [7,12], and whole cells [7,13]. Transducers measure molecular interactions taking place on the bioreceptor and output an electrical signal based on that interaction. These include electrochemistry [7,8], mass sensitivity [7,14], optical sensing [7,15,16], and thermal sensing [7,17]. Most published SNO detection methods rely on optical methods in the form of UV-visible detection or more commonly chemiluminesence by first degrading SNOs into NO. The NO is then exposed to ozone to yield nitrogen dioxide in an excited state (NO_2_*). When this excited NO_2_* relaxes back to its ground state it emits light in the red and near infrared region [18]. These methods have a limit of detection too high to make them useful for detecting SNOs at their normal biological levels.

**Fig 1:**
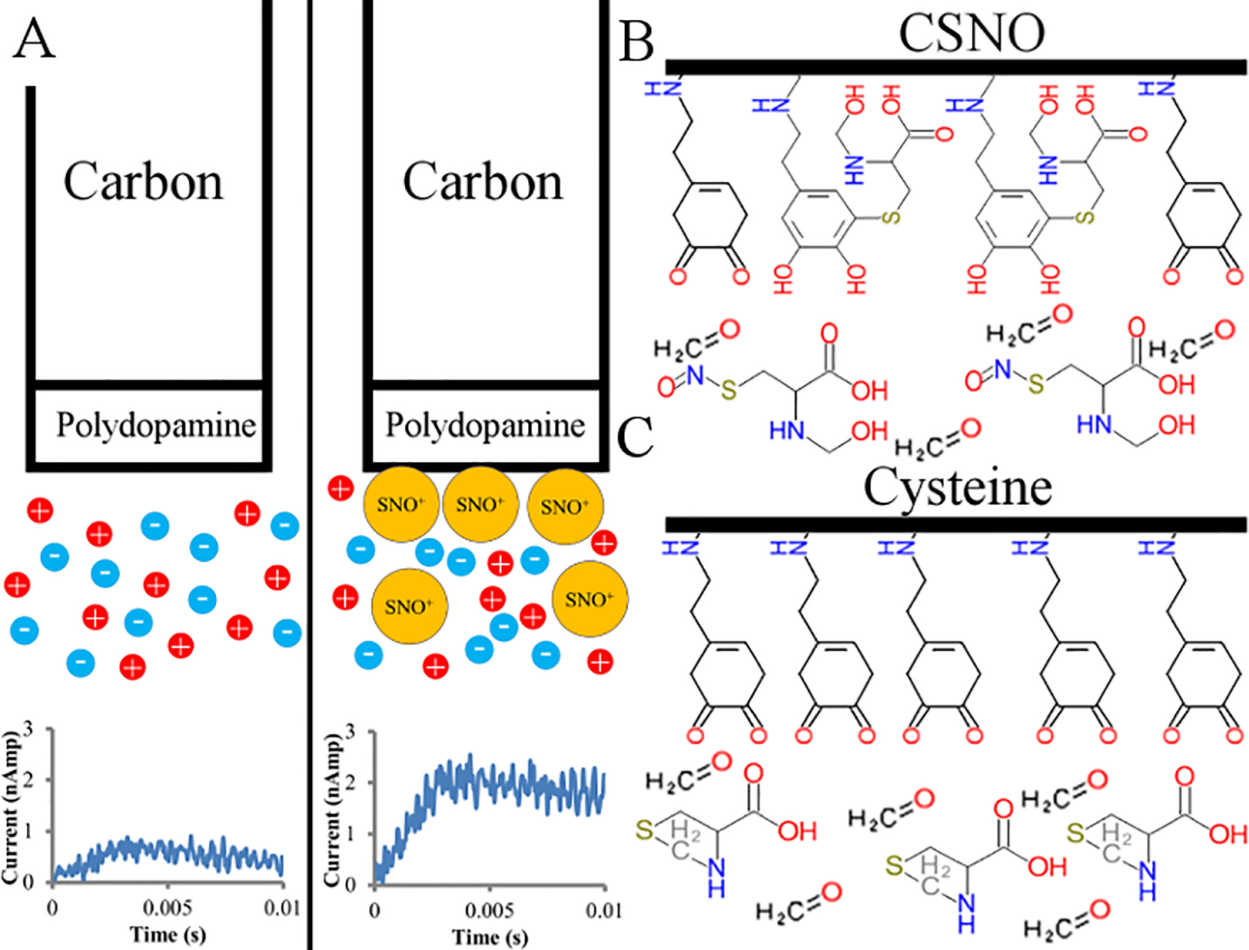
An overview of capacitive sensors. (A) A schematic of a capacitive sensor. (B) The proposed chemistry with CSNO. (C) The proposed chemistry with L-cysteine. CSNO can interact with quinone surface of the polydopamine, while formaldehyde converts cysteine into thiazolidine, which cannot interact [21, 22].

Field-Effect Transistor (FET) capacitive biosensors can detect trace amounts of specific biochemicals in the complex milieu of biological samples [8]. They work by using an electrical circuit and then measuring the change in capacitance of that circuit as it interacts with the molecule of interest (Fig 1A) [19]. This requires the capacitor in the system to be made functional with some molecule that specifically interacts only with the analyte of interest, and is most often done by employing antibodies against a specific molecule in order to measure the antibody-antigen interaction [20]. In many cases a high quality antibody to the analyte of interest is not available and other chemical means must be used. The functionalized surface of a FET biosensor is coupled to a semiconductor within the capacitor to ensure a change in the net charge of the functionalized surface will cause a significant change in the capacitance of the semiconducting layer beneath it [8]. This allows for the detection of trace molecule in a biological solution, and when semiconducting materials are employed give them a limit of detection determined by the strength of the interaction between the functionalized layer and the analyte.

Dopamine is an organic catecholamine, which under oxidizing conditions forms the melanin polymer, polydopamine [23]. Polydopamine forms thin layers on surfaces when it oxidizes and has a number of useful properties including semiconducting properties and a highly reactive surface [24,25]. This surface will attack any free thiol or primary amine in solution with it, covalently bonding them to the polymer surface [21]. We will also present evidence that the polydopamine chemically reacts with SNOs in a manner unique from their parent thiols, allowing us to covalently bond to SNOs in a solution of polydopamine. Chemically bonding additional molecules to a polydopamine surface, significantly changes its conductive properties making a polydopamine layer ideal for both the semiconducting and the functionalization layer of a SNO-biosensor [24]. However, there would need to be a way to prevent unrelated free thiols and amine groups, which are prevalent in biological samples from interacting with the polydopamine layer during experiments.

Formaldehyde is a commonly used chemical which will block all primary and secondary amines in a solution by means of the Eschweiler–Clarke reaction, as well as blocking all free thiols. This reaction works by methylating all primary amines, secondary amines, and free thiols according to the following reactions [22,26,27,28].

$$R-NH_{2}+CH_{2}O↔R-NCH_{2}+H_{2}O$$

$$Cysteine+CH_{2}O↔Thiazolidine+H_{2}O$$

Formaldehyde is used to preserve biological tissues and has the useful feature of not being able to interact with the S-NO bond in SNOs. Methylating all primary amines, secondary amines, and free thiols blocks covalent binding to catecholamine ring in polydopamine and hence prevents interactions of the polydopamine surface with these compounds. This means that if a biological sample is pretreated with formaldehyde, the polydopamine surface will not sense free thiols or amines, and will specifically sense nitrosylated thiols (Fig 1B and 1C). If this chemistry holds true, it should mean that we will detect SNOs in a solution of biological materials under conditions where they exist, and that we will be able to abolish that detection under conditions that degrade SNOs into NO and their parent thiols.

In this paper, we will provide the method and technical specifications for building and running a SNO-specific capacitive biosensor. We will provide data about its limits of detection, failure modes, and potential applications for sensing SNOs in biological samples. We will show that our method detects minute quantities of SNOs, does not detect parent thiols in high concentrations—and does not give a signal in biological samples after application of methods, which specifically degrade SNOs. This sensor will allow better detection of SNOs in a variety of biological systems.

## Methods

### Human studies

The Review Board giving permission for the human studies was the University Hospitals Institutional Review Board. The details are: PI: Dr. James Chmiel. Title: Blood Collection for Research Related to Asthma, Cystic Fibrosis and Other Pulmonary Disorders. ID Number: IRB# 06-13-08. Animal Studies: None.

### Materials

Unless otherwise specified all reagents were obtained from Sigma-Aldrich. Carbon fiber electrodes were obtained from ALA Scientific (CFE-2). All buffers were made the day of the experiment in doubly deionized water. This is to prevent formaldehyde degradation in running buffer. All experiments were run in one of the following buffers. Plating Buffer: 10 mM Potassium Monobasic Phosphate Buffer adjusted to pH 7.5 using NaOH with 1 μM CuCl_2_. The copper in solution enhances polydopamine’s semiconducting properties [24,25]. Running Buffer: 10 mM phosphate buffered saline, pH 7.4 with at least 0.8% formaldehyde. It is very important to use phosphate buffered saline that is low in metal contaminants, as copper and iron contamination will degrade SNOs and cannot be easily removed as most metal chelators are neutralized by formaldehyde. It is suggested to purchase low metal concentrated PBS to make running buffer.

### Sample preparation

Biological samples underwent centrifugation (30 sec at 14,000 rpm) to remove large aggregates and then separated using a 10 kDa spin filter (2 min at 12,000 rpm) to remove all large proteins and other particles. In particular, this would remove all SNO-degrading enzymes and all Cu binding proteins. There is no significant concentration of free Cu(II) in the blood serum we use for detection and hence no Cu-mediated degradation of SNOs is likely.

The low mass fraction was collected, and two aliquots (100 μL each) were flash frozen with dry ice in ethanol. The first aliquot of 100 μL was diluted into 10 ml of running buffer and allowed to react at room temperature for 15 min. It was then run on the sensor. The second aliquot was incubated under a UV light and spiked with 3 mM HgCl_2_ for 90 min to degrade all SNOs in solution. Afterwards, the sample was diluted into 10 ml of running buffer and incubated for 15 min before being run on the sensor. This negative control is essential to run with all biological samples to ensure there are no non-specific interactions with chemicals inside of the biological sample. If the negative control samples give a positive result, the concentration of formaldehyde in the running buffer should be increased to block all free amines and thiols.

### Protocol

**Fig 2:**
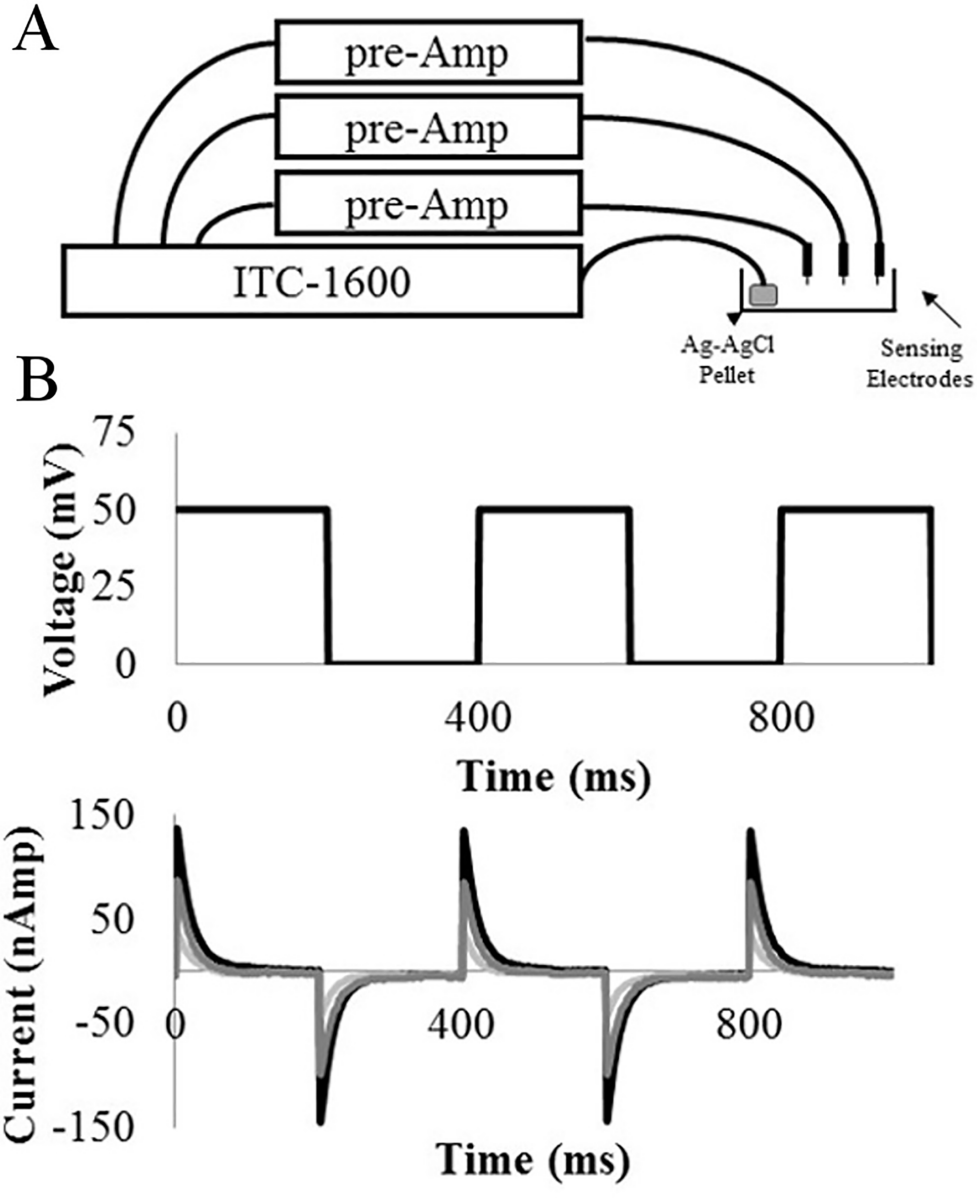
A schematic and example data of the sensing rig. (A) A schematic of the setup. (B) Example data trace showing the input voltage and the current response.

Up to 3 functionalized electrodes were attached to three separate pre-amplifiers (SR560, Stanford Research), which, in turn, were connected to three separate AD channels of an ITC-1600 (HEKA Corporation) (Fig 2A). The entire setup was enclosed in a well ventilated Faraday cage to block out all ambient electrical noise. Current injection was provided by connecting a small Ag-AgCl ground pellet (E205, Warner Instruments) to a DC channel of the same ITC-1600. All pre-amplifiers were set to ground coupling and a 10x gain. The 3 electrodes were suspended above a 10 ml petri-dish such that the tips of the carbon electrodes would be submerged in running buffer when the petri-dish is filled. Once all 3 electrodes are positioned and attached to their pre-amplifiers and the ground pellet is placed in the petri-dish and connected to the ITC-1600, the petri-dish can be filled with running buffer and the circuit completed. Once the dish is filled, the pre-amplifiers should be set to DC coupling and the experiment can begin. It is very important that the pre-amplifiers must be set to ground coupling while the dish is filled so that current spikes caused by flowing saline near the electrodes before the circuit is fully formed do not damage the pre-amplifiers or the electrodes.

All experiments were performed at room temperature with no stirring of the solutions. Once all electrodes have their tips submerged in running buffer, the controls for the sensing experiment can begin. A single sensing experiment is conducted by applying a step potential across the electrodes using the following protocol. A step potential was applied across the electrodes by first stepping the potential to 0 mV, holding it for 200 ms and then stepping it up to 50 mV and holding it for 200 ms. This process was repeated for 30 sec or for a total of 75 repetitions of the step potential. The resulting current traces for all three electrodes via their pre-amplifiers were recorded simultaneously on three separate channels of a ICT-1600 data acquisition unit (Fig 2B). A higher step potential can be used for electrodes that do not show a strong enough response to stimulation but a step potential of 150 mV or higher should never be used to avoid damaging the sensing electrodes. This represents a single recording during an experiment. A recording is taken before the experiment begins to ensure all electrodes are in good electrical contact with the running buffer. Then the system is perfused with 10 ml of running buffer and a Baseline recording is taken. The system is again perfused with 10 ml of running buffer to mimic a blank sample injection and a Blank Injection recording is taken. Afterwards the system is perfused with 10 ml of running buffer to mimic a washout step and a recording is taken. The raw data should be reviewed at this time to ensure there is no signal drift or other artifact in the data. If drift or artifact are observed, the data should be discarded and the Baseline, Blank Injection, and Blank Washout steps repeated until a stable baseline is obtained. Once the system is shown to have a stable baseline reading, the sample, prepared as described above, should be injected and allowed to incubate for 2 min before a Sample Injection recording is taken. The final injection volume is 100 μL of sample diluted into 10 ml of running buffer. Finally, after 4 min of total incubation time, the sample should be washed out of the petri-dish by injecting 20 ml of running buffer, and a final Sample Washout recording taken.

Immediately after the Sample Washout recording is taken, the electrodes should be removed from the petri-dish. The dish and its running buffer should be discarded and replaced with a fresh petri-dish. This is refilled as above to prepare for a new experiment. A single set of functionalized electrodes should not be used for sensing experiments more than 5 times in a row before being re-functionalized to prevent saturation of the polydopamine surface. Exposing electrodes to high concentrations (~nM to mM) of SNOs will saturate the electrodes after a single experiment, while samples without any SNOs will not saturate the electrodes at all. After electrodes have been removed from the old solution, the resulting data should be saved and processed as described below. The time that parylene coated electrodes sit in running buffer should be minimized as aqueous solution will slowly dissolve the parylene coating, creating pinholes in the insulating coating.

### Absorption spectroscopy

All absorption spectroscopy experiments were performed using a SpectraMax Plus 384 Plate Reader (Molecular Devices) with a standard 96 well plate (Costar, #3596). We mixed running buffer alone, 100 μM dopamine hydrochloride in running buffer, 100 μM S-nitroso-L-cysteine (CSNO) in running buffer, and 100 μM dopamine hydrochloride and CSNO in running buffer and allowed all four samples to incubate for 15 min in the 96 well plate before a spectrum reading was taken between 350 nm and 750 nm in 5 nm increments.

### Mass spectrometry

All mass spectrometry was performed using a Thermo Finnigan LCQ Deca. We prepared 100 μM dopamine hydrochloride in running buffer, 100 μM CSNO in running buffer, and 100 μM dopamine hydrochloride and CSNO in running buffer and allowed all four samples to incubate for 15 min in Eppendorf tubes. Afterwards we directly infused 100 μL of each solution onto the mass spectrometer and recorded the resulting m/z range between 50 and 500 m/z for 18 s. The resulting mass spectra were averaged over the 18 s window and the averaged spectra were analyzed.

### Statistical analyses

All statistical analysis was done using Microsoft Excel 2016. To determine statistical significant differences from blank injections, we employed the Two Tailed Student’s T-test, assuming a heteroscedastic distribution. Only differences with a p-value of less than 0.01 were considered to be significantly different from blank injections. All average normalized charge responses are presented with their mean value followed by the standard error.

## Results

### Interaction between SNOs and dopamine

**Fig 3:**
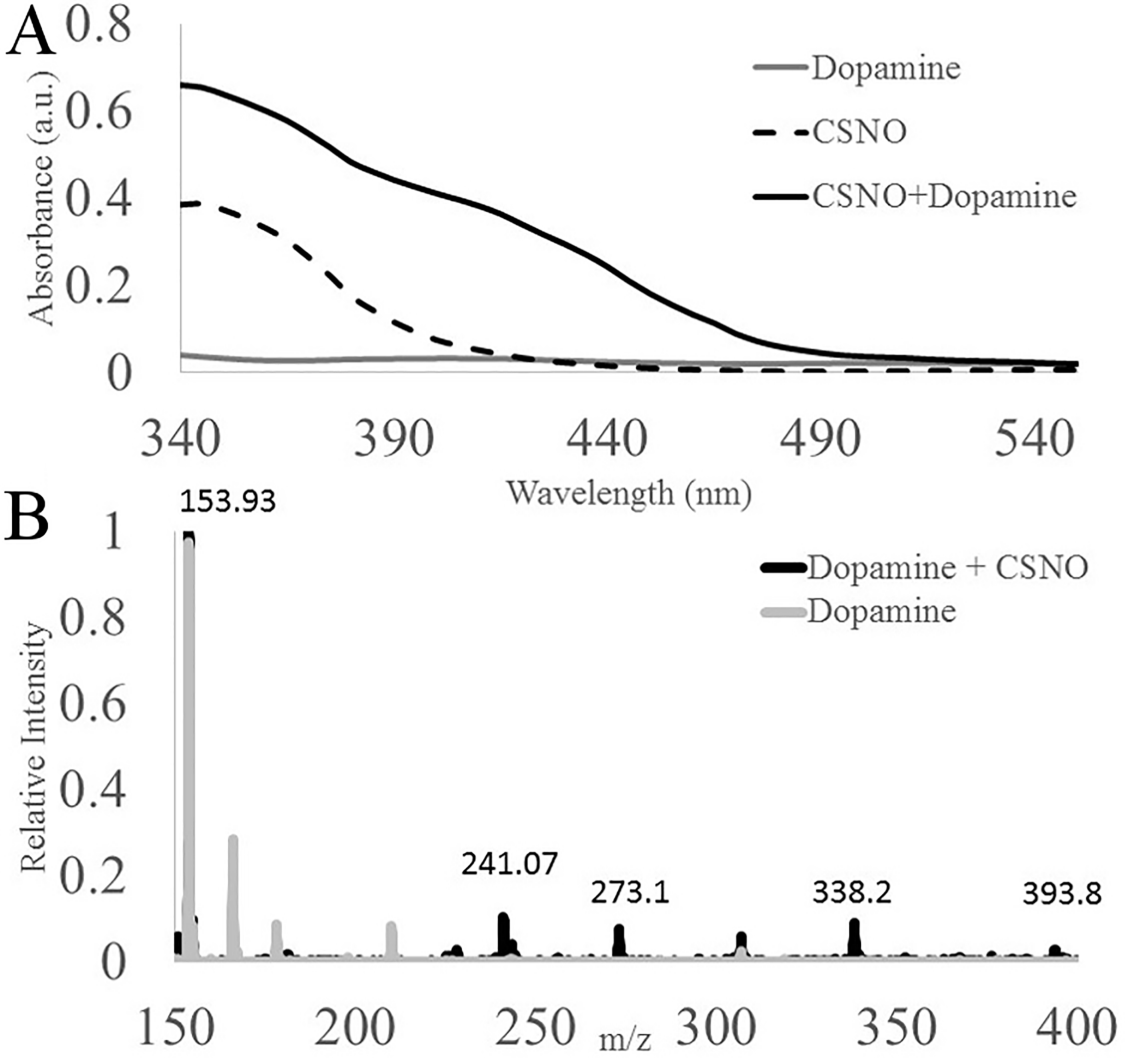
The interaction between dopamine and CSNO in solution. (A) The Absorption spectra of a dopamine, CSNO and dopamine + CSNO in running buffer. (B) Mass Spectra of either dopamine alone or CSNO with dopamine.

We measured the interaction between CSNO and dopamine by absorption spectrometry and mass spectrometry (Fig 3). First we incubated 1 mM CSNO in running buffer for 15 min before adding equimolar dopamine for an additional 15 min. After that time, absorption spectra were taken of CSNO alone, dopamine alone, and CSNO + dopamine. Dopamine itself showed little absorbance between 340 and 550 nm, while CSNO showed a strong absorption around 340 nm. When mixed with dopamine, CSNO shows a stronger absorption at 340 nm, while gaining an absorption peak centered around 420 nm. This was further characterized by direct injection mass spectrometry. Here, we combined dopamine and CSNO in distilled water and incubated them for 15 min. We observed that this mixture formed di-sulfide cysteine or caused dopamine’s mass to shift (Fig 3B). The m/z peak 153.9 represents unreacted dopamine, 241 represents cystine, 273.1 represents dopamine covalently bound to 1 cysteine molecule, 338.2 represents dopamine covalently bound to 1 cysteine molecule and 1 formaldehyde molecule, 393.8 represents dopamine covalently bound to two cysteine molecules. All other peaks in this spectrum are contaminant peaks.

### Dose response for limit of detection

**Fig 4:**
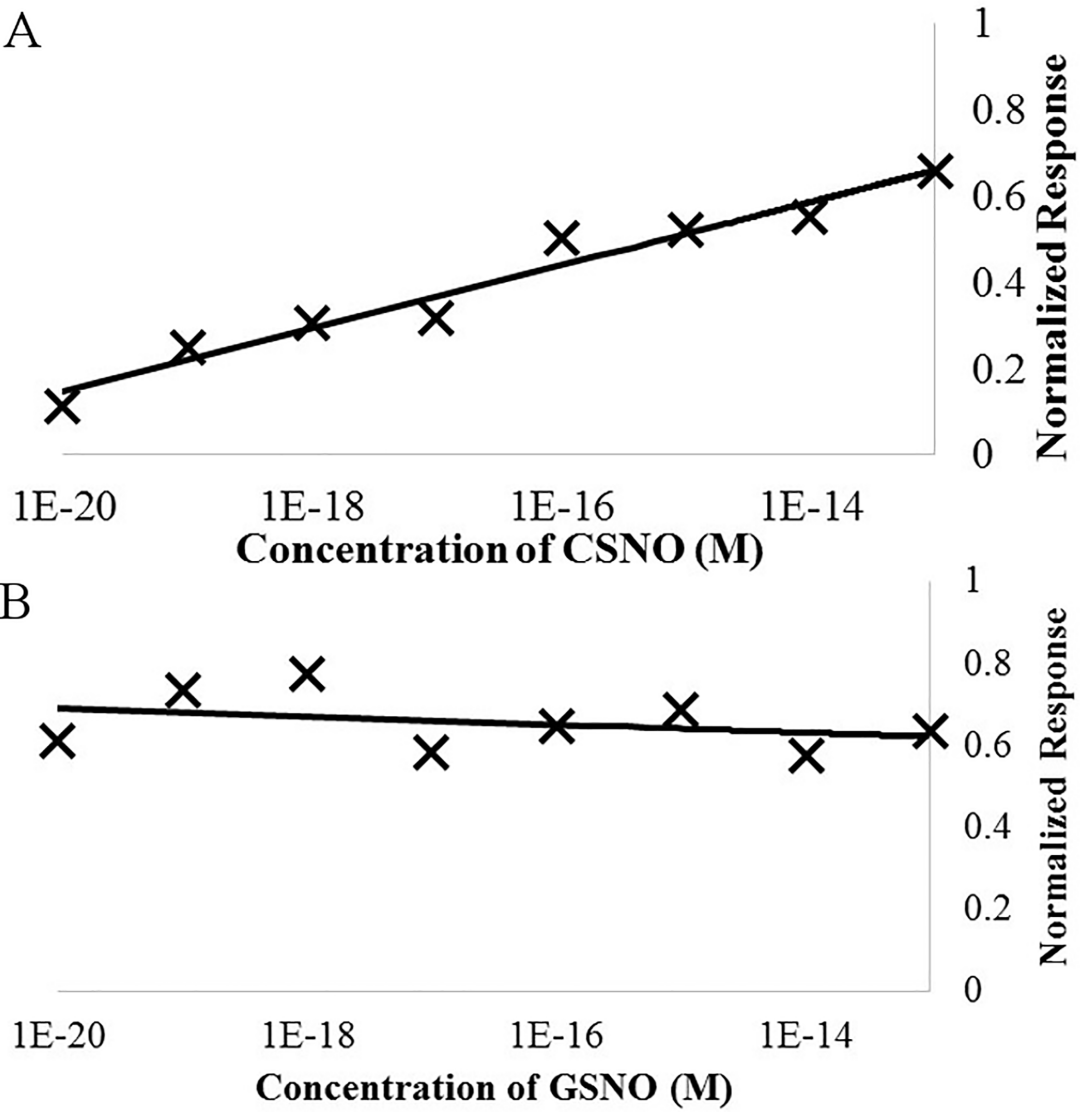
The dose response of sensors to SNOs. The average normalized response of the sensing electrodes after incubation with increasing concentrations of (A) CSNO, and (B) GSNO.

Functionalized sensing electrodes were tested for quality and then exposed to increasing concentrations of CSNO or S-nitroso-L-glutathione (GSNO) to test the relationship between small molecule SNO concentration and the normalized response of sensing electrodes to that compound (Fig 4). See supplemental section (S1 Appendix) for a full description of the normalized response but in brief it is a number the represents the change in charge accumulation of the sensing electrode after a blank injection or a sample injection. This number is 0 for all times when the blank charge accumulation is larger, and ranges between 0 and 1 for times when the sample injection charge accumulation is larger. When running buffer in injected in place of a sample (Blank Injection), the electrode gives an average response of 0.030 ± 0.065. Samples that contain a saturating concentration of SNOs, give an average response of 0.65 ± 0.10. In general, individual electrodes have a high amount of variability with regards to the signal they produce, but a much more stable signal emerges when multiple electrodes are run in parallel and the results are average together.

By averaging the results of many separate sensing electrodes, we revealed a log-linear dependence between the concentration of CSNO and the normalized response (Fig 4A). The correlation equation from fitting this data is r = 0.0319lnC + 1.642 (R^2^ = 0.9575), where r is the normalized response and C is the molar concentration of CSNO. The limit of detection (LOD) for CSNO is calculated to be 1.25 x 10^−19^ M, or 0.125 aM. CSNO concentrations greater than 100 fM saturate the sensing electrode’s response and do not produce a linear response with concentration. This is a marked improvement over published SNO sensors, which at best have a LOD of 50 nM [6, 29,30]. We also added increasing concentrations of GSNO to functionalized sensing electrodes and measured the response (Fig 4B). We found that in contrast to CSNO, these sensing electrodes had no linear dependence with respect to concentration, and immediately saturate at a concentration of 1 x 10^−20^ M, or 0.01 aM GSNO. Upon further investigation, the normalized response to GSNO jumps from 0.029 ± 0.021 at 1 x 10^−21^ M GSNO to 0.612 ± 0.083 at 1 x 10^−20^ M GSNO. This makes the effective LOD for GSNO 1 x 10^−20^ M, or about 60 molecules of GSNO in 10 mL of running buffer. This means that the concentration of GSNO can be determine within a biological solution to within one log order by performing a serial dilution study to determine when the signal appears. It also means that it is possible to determine the difference between CSNO and GSNO in solution by seeing if the signal gradually fades with dilutions or suddenly vanishes.

The tepid response to CSNO compared to GSNO can also be partially explained by the relative stability of CSNO and GSNO in Running Buffer. We incubated 1 mM of either CSNO or GSNO in running buffer and monitored its stability by means of absorption at 340 nm. After 15 min of incubation in Running Buffer, only 59% ± 2% of CSNO added to the buffer remained in solution, while 90% ± 4% of GSNO added to the buffer remained. This degradation of CSNO and no GSNO is likely a combination of trace heavy metal contamination and pH degradation due to the relatively alkaline pH of our Running Buffer. SNOs that degrade during the incubation step will be blocked by the formaldehyde of the running buffer and hence will not interact with the sensing electrode. This most likely means that at ultra-low concentrations of 1 zM, the sample fully degrades before interacting with the sensing electrode.

### Specificity of detection of SNOs

**Fig 5:**
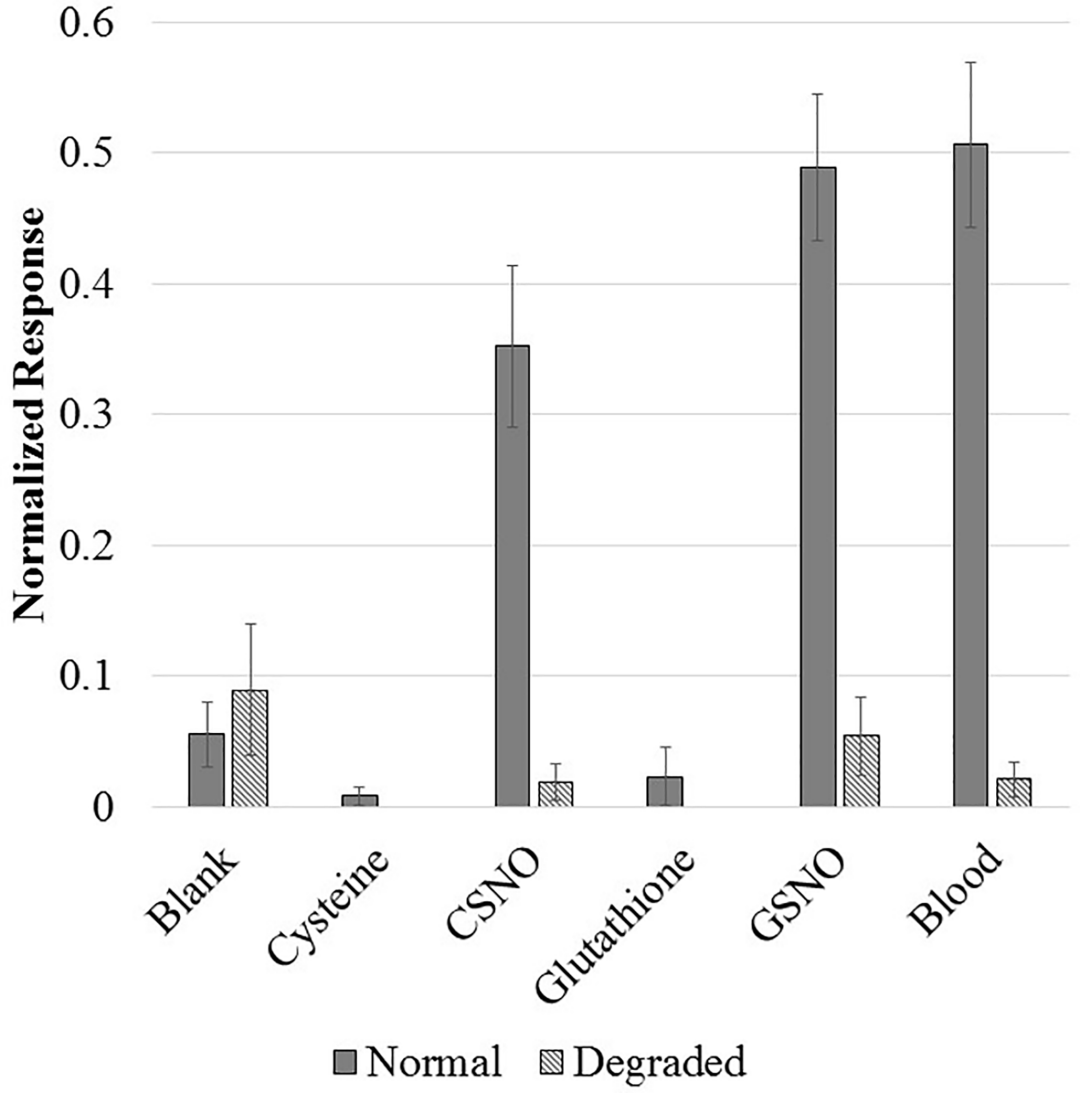
The selectivity for SNOs. The average normalized response of the sensing electrodes after incubation with either: running buffer, 100 μM L-cysteine, 100 pM CSNO, 100 μM L-glutathione, 100 pM GSNO, or venous blood plasma. These samples were either prepared normally or preincubated with mercurous chloride and exposed to UV light to degrade all SNOs in solution.

We prepared samples of CSNO, GSNO, L-cysteine, L-glutathione and human venous plasma in the manner described in Sample Preparation, and ran each of these samples either: immediately or after pre-incubating them with of 3 mM HgCl and 90 min of exposure to UV light. UV light and HgCl are shown to degrade SNOs and as such should produce a negative signal [31]. Solutions of CSNO and GSNO were prepared by diluting a stock solution of the SNO to 10 nM in distilled water and then mixing 100 μL of the stock with 10 ml of running buffer for a final, in petri-dish concentration of 100 pM SNO. The human plasma was prepared in an identical fashion to the stock SNO solutions. The resulting normalized response was recorded for each of these fluids (Fig 5). Blank injections of 100 μL of distilled water and running buffer were also performed at the same time to ensure stability of the sensing electrodes. We prepared a 10 mM stock of cysteine or glutathione, and then injected 100 μL of the stock in 10 ml of running buffer for a final concentration of 100 μM. We did not prepare cysteine or glutathione under degrading conditions as neither has a potential S-NO bound to break. Of the samples prepared under non-degrading conditions only the CSNO, GSNO, and blood samples gave a significant (p < 0.01) signal over that of a blank solution. Cysteine and glutathione samples showed no significant differences from blank buffer injections showing that while the sensing electrodes will react to trace concentrations of SNOs, they are insensitive to high concentrations of their parent thiols. No sample prepared under degrading conditions produces a signal significantly different from blank injections, strongly suggesting that the signal from the venous blood plasma was originally due to the presence of SNOs.

### Sensitivity to changes in buffer

**Fig 6:**
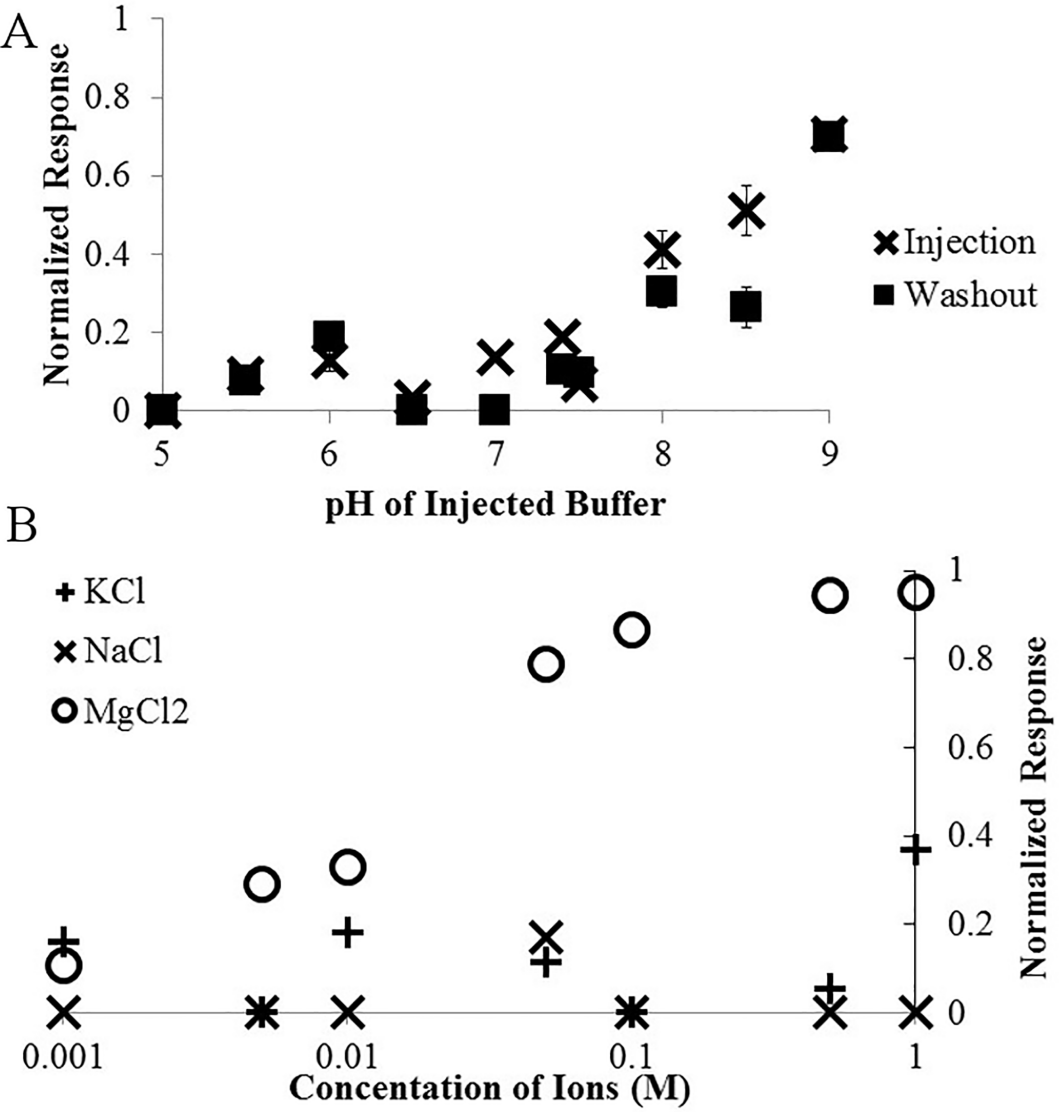
The response to changes in solution. The average normalized response of the sensing electrodes to blank running buffer injections of varying pH (A) or with various concentrations of potassium, sodium, or magnesium (B).

We injected blank running buffer at different pHs in place of real samples and recorded data both when the pH of the solution had shifted and then again once the pHed buffer had been washed out with 20 ml of normal running buffer. We calculated a response ratios and performed a Student’s T-Test between all of the various pHs and blank buffers. We found that while alkalizing the running buffer does cause a slight false positive, it does not statistically significantly change (p < 0.01) the response after the sample is washed out until the pH of the buffer is raised to 9.0 (Fig 6A) This pH is destructive to S-nitrosothiols and would never be used in a laboratory setting. Acidifying the running buffer did not generate any false positives, but did affect the sensor by decreasing the inherent random drift that the sensor experiences, and hence slightly lowered the response. This was not statistically significant though with a p-value of 0.17 for pH 5 washout and 0.75 for pH 5.5.

We mixed various concentrations of stock solutions of potassium, sodium or magnesium into running buffer normally and injected it in place of a real sample (Fig 6B). Sodium gave no discernable signal, while potassium gave a significant (p < 0.05) signal at 1 M KCl. Magnesium did give a statistically significant false positive signal at concentrations of 5 mM MgCl_2_ or higher. While this could pose a challenge to some experiments, the basal level of plasma magnesium levels has been reported to be 1 mM, much less than a level which would cause a significant false positive [32]. Finally, should magnesium cause a false positive in lab tests, this can be resolved by simply further diluting the sample to ensure the injected sample has a concentration of magnesium lower than 5 mM. The relative insensitivity of the sensing electrodes to changes in ionic strength can be explained by the composition of the running buffer. As it is phosphate buffered saline, it already has a rather high ionic strength and hence small changes in the concentrations of ions, which would come from biological samples are unlikely to affect the real signal.

## Discussion

### Biological significance

**Fig 7:**
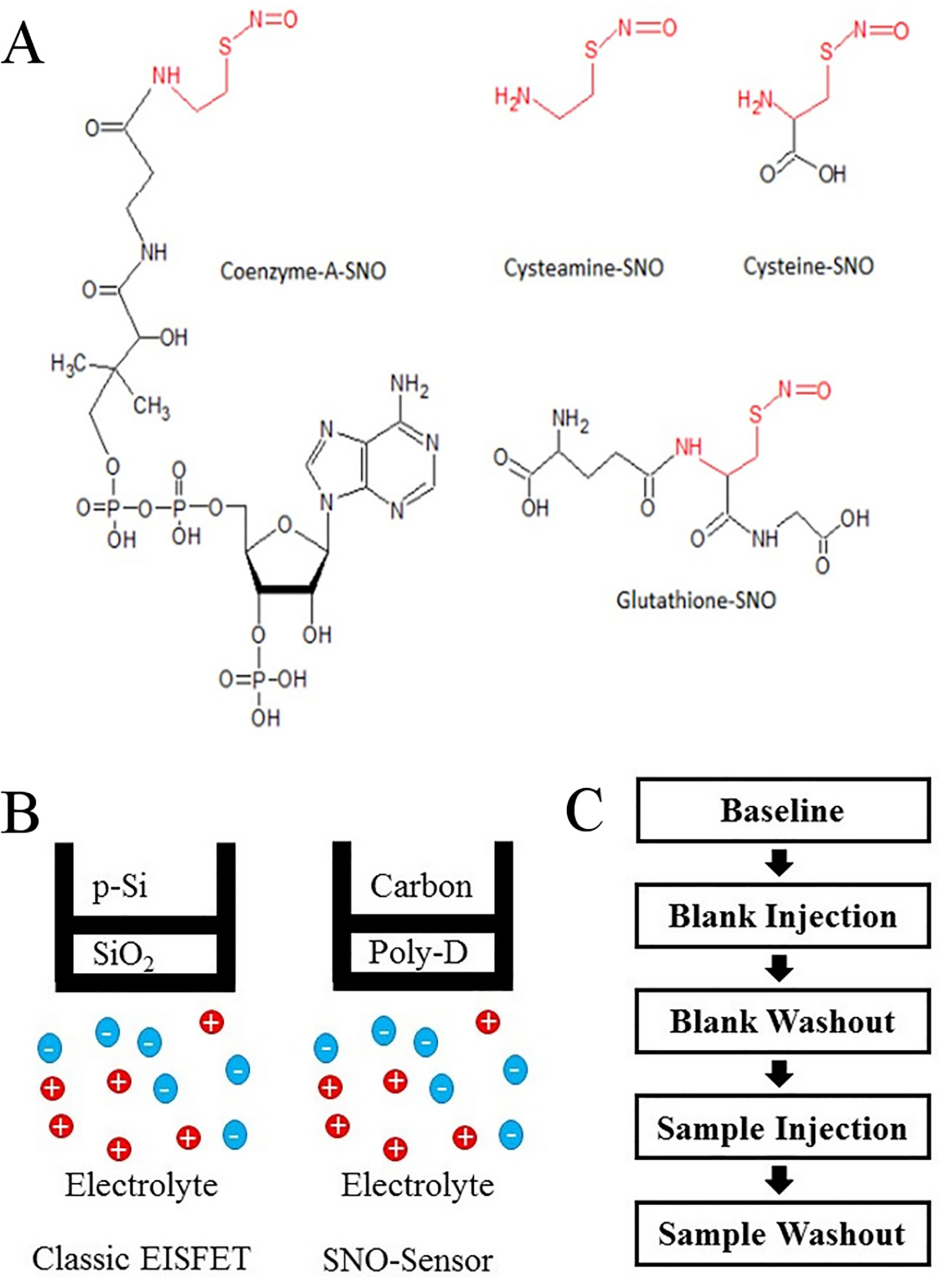
Molecular and experimental diagram. (A) Diagrams of endogenous small molecule SNOs, (B) Schematic of the classic EISFET compared to the polydopamine coated sensor used in SNO detection, and (C) the data collected during a single sensing experiment.

The biological role of NO addition to heme groups in soluble guanylyl cyclase (sGC) and in hemoglobin has been understood for nearly two decades [1]. NO addition to protein thiols is now also known to be an important signaling reaction – termed S-nitrosylation – that is analogous to phosphorylation, glutathionylation, palmitoylation, acetylation and other physiological protein modifications [33]. S-nitrosylation occurs downstream of cellular NO synthase (NOS) activity [33] and through through intermediate, endogenous low molecular weight SNOs (Fig 7A). These latter, low molecular weight SNOs are endogenous, and the metabolism of each is regulated by specific enzymes [1,33–35]. There are many examples demonstrating that this type of signaling occurs across a broad range of biological systems (Table 1, [1,36]). Disorders of protein S-nitrosylation are relevant to the pathophysiology of many diseases [1,33,34, 37], and S-nitrosylation is emerging as a field relevant to many biological disciplines [1] (Table 1). In addition, intermediate low molecular weight SNOs (**Fig 7A**) appear to act as ligands in many signaling reactions. However, current assays for S-nitrosylated proteins lack sensitivity (Table 2) and often used near the limit of detection [6], which hampers translational research progress.

**Table 1:** Examples of the role of S-nitrosylation in biology and medicine.

Discipline	Examples
*Prokaryotic biology*	Cell biology
	Infectious disease
*Plant biology*	Cell signaling
*Mammalian biology*	Cell signaling
- Neuronal	Parkinson’s disease
	Alzheimer’s disease
	Central apnea
	Amyotropic lateral sclerosis
-Muscular	Heat stroke
	Fatigue
	Muscular dystrophy
- Cardiovascular	Myocardial infarction
	Stroke
	Shock
	Arrhythmias
-Respiratory	Pulmonary hypertension
	Asthma
	Cystic fibrosis
	Altitude adaptation
	Bronchopulmonary dysplasia

**Table 2:** Currently available assays for S-nitrosothiols.

Assay	S-^15^NO detected	Limit of detection	Drawbacks
ELISA/spectrophotometry	No	500 nM	Insensitive
Chemiluminescence	No	50 nM	Poor reproducibility
Mass spectrometry with/without biotin switch	Yes	250 nM	Insensitive; artifacts in sample preparation.
Immunodetection using anti-S-nitrosocyteine antibodies	No	1 mM	Antibody is not sensitive or specific
Cavity ring-down spectroscopy	Yes	500 nM	Expensive, labor-intensive

It should be emphasized that S-nitrosylation is as a regulated cellular process, rather than a non-specific toxicity. Many proteins catalyze the formation and degradation of protein SNO bonds. NOS activity can result in localized S-nitrosylation of co-scaffolded proteins, conventionally at cysteine S-nitrosylation motifs (Fig 7A) [38]. Protein S-nitrosylation is also catalyzed by enzymes other than NOS [39]. Note that protein denitrosylation is also enzymatically regulated; indeed, the kinetics of this denitrosylation can represent a major obstacle to accurate measurement in biological samples. However, a majority of protein S-nitrosylation-denitrosylation reactions appear to involve the formation of GSNO and other intermediate, low-mass SNOs. S-Nitrosylation reactions are involved in the full spectrum of cell signaling functions. They regulate epigenetic effects [40]. S-Nitrosylation can regulate the expression of nuclear regulatory proteins, including NFκB, hypoxia-inducible factor (Hif) 1 and specificity proteins 1 and 2 [41]. S-nitrosylation affects the activity of membrane-associated proteins and degradation of many proteins [2].

There is emerging evidence that disorders of cellular processes described above are observed in a variety of pathophysiological processes ranging from cancer to Parkinson’s disease (Table 1). These disorders are major causes of morbidity, mortality and increased health care costs world-wide [1,33,34,36,42]. The clinical translation of these findings has been severely hampered by the lack of a reliable, sensitive assay [43]. We anticipate that this improved assay for GSNO has the capacity to transform management of diseases involving virtually every organ system. In many disease states, circulating or tissue levels of low mass-SNOs are abnormal [1]. For example, they are low in severe, life-threatening asthma [44] and high in life-threatening septic shock [45]. The problem is that the limit of detection for these molecules using current technology is mid-nM [1,45,46,47]. In many tissues, normal levels are at or near the limit of detection, and in disease states with increased catabolism [1,44], “low” often means “undetectable.” There is universal agreement that a more sensitive assay is needed [2]. It is clear that aM sensitivity of our capacitance method is more than needed but it certainly represents an important step forward in SNO-detecting technologies.

### Limitations of current assays

Photolysis-chemiluminescence and reduction-chemiluminescence methods (Table 2) can be sensitive down to 5 pmol (50 nM for a 100 μL sample injection) [6,29,30]. However, even this, the most sensitive type of assay, is often used near the limit of detection in biological samples, making it difficult to distinguish signal from noise; this creates significant problems with reproducibility [30]. The Meyerhoff laboratory has developed a selenium-based and related electrochemical sensors for SNOs in blood. This sensor is somewhat less sensitive (limit of detection about 20 nM) than ours (limit of detection around 0.125 aM) and the utility at physiological pH may not be as optimal [48,49] Liquid chromatography/mass spectrometry following biotin substitution is the favored proteomic method for measuring S-nitrosylated proteins, but requires many preparatory steps which can disrupt, or artifactually form, SNO bonds, and it lacks sensitivity [29]. All other assays are only sensitive to ~250–500 nM, often above the normal concentration in biological samples [1,6,50]. More recently, reduction coupled to cavity ring-down spectroscopy has been developed as a SNO assay [6]; this assay can sensitively distinguish ^14^NO from ^15^NO SNOs, but has no other advantage over reduction-chemiluminescence and is substantially more cumbersome and expensive.

Intracellular SNO bonds are stabilized by steric sequestration in proteins and by localization in membranes or vesicles [51,52]. When cells are lysed, enzymatic and inorganic denitrosylation begins and the assay signal begins to be lost [30]. Different SNOs vary in stability [53], and trans-nitrosation can convert stable S-nitrosoproteins to species that are labile in the intracellular environment [51,53]. Non-enzymatic denitrosylation after cell lysis or *ex vivo* is favored by copper and iron ions, and by light, heat, ascorbate, bilirubin and sulfite [29]. Sample manipulation with exogenous reducing agents (such as dithiothreitol)—or even gel electrophoreses—can break SNO bonds [30]. Thus, endogenous SNOs can be denitrosylated *ex vivo* before being assayed. Moreover, SNOs can be formed artifactually from environmental nitrite at low pH [30]. Thus, isolation and measurement of SNOs can artifactually break or form SNO bonds.

The method we present here has some advantages over existing SNO detection methods and most importantly, in sensitivity and in the use of formaldehyde as a blocking agent. The artificial creation of SNOs (generating of SNOs from free thiols and free nitrites in solution) by detection methods has been a bane of the field [18]. As almost all biological samples have free nitrites, great care must be used to not inadvertently convert those nitrites into nitric acid, and thus generate SNOs during sample preparation. By covalently blocking all free thiols during the sample preparation step, we make generating artificial SNOs from free nitrites in solution impossible, leaving only biologically relevant SNOs. This when coupled to our limit of detection which is far below all relevant biological SNO levels makes this method ideal for studying the role of SNOs in both normal biology and disease models.

### Potential uses for the capacitive SNO sensor

No other capacitive biosensor has been developed to measure SNOs, so there is not prior art with which to compare this new sensor. This is largely because there is not a good antibody against low-mass SNOs to permit antibody-antigen-based signaling [38]. There has also not previously been a chemical method for selective SNO measurement using a capacitive sensor. Our method may prove vital to furthering the SNO field. To name a few examples, we will focus on asthma, cystic fibrosis, fatalities to Ebola virus, and locating the source of endothelium-derived relaxing factor L-CSNO. It has been previously published that airway SNOs are much lower in children with asthma than with normal children [44]. It is possible and useful to show that exercise stimulates the production of SNOs, particularly GSNO in human subjects. To accomplish this, we would need to ultra-high sensitivity of our novel method, as samples taken from children would need to be small in volume in order to avoid harm to human subjects. In cystic fibrosis, SNOs have been shown to increase expression of mutant CFTR in rats [54], and it’s possible that endogenous SNO production could become an effective treatment for children with cystic fibrosis. There have also been multiple studies of the differences between fatal and non-fatal cases of Ebola virus in African populations, and it has been reported that the main indicator of death from the Ebola virus was elevated blood nitric oxide levels [55]. This almost certainly corresponds to elevated levels of blood SNOs, but to study this effect we must have a method capable of sensitively measuring spikes in very low levels of SNOs in normal patients and more importantly detection when levels of SNOs begin to elevate. Finally, this method can be employed in the location of endogenous SNOs, which are stored in vesicles throughout the body. An ultrasensitive method can be used to detect vesicular release from plated primary cells as well as in fluids collected from tissue or even whole animal preps. The volume of a single neuronal vesicle is, on average, 3.2 x 10^−20^ L [56]. Assuming a vesicle SNO concentration of 100 mM, this would result in a final in petri-dish concentration of 3.2 x 10^−19^ M, or 0.32 aM, just at our LOD. This would allow us to theoretically detect the release of individual vesicles of SNOs from a single isolated cell, opening up all manner of avenues of research into the natural of their release and their role in normal biological function.

All of the above mentioned potential studies require a method of SNO detection more sensitive than the current mid-nM LOD that previously published methods provide. While some of the do not require high zeptomolar sensitivity, they all benefit from this LOD by allowing multiple experiments to be performed with small initial sample volumes. The quantity of GSNO in solution may not be able to be precisely determine, but by performing serial dilutions it can still be determined to within a half-log order of concentration in biological samples, and detecting changes in SNO concentrations can easily be determined by beginning experiments at a dilution factor that abolishes the control SNO signal, or in the case of SNOs being down-regulated, by determine how much more concentrated the starting sample needs to be before the signal reappears. Furthermore, the difference in behavior of CSNO and GSNO (the two primary endogenous SNOs) allows for us to distinguish them in biological solutions by studying the reaction of the sensing electrodes to the biological sample, and suggesting to us what SNO we are detecting in solution.

### Sensitivity and limitations of FET biosensors

Label-free electrolyte-insulator field-effect transistor (EISFET) biosensors have come to prominence in the past decade for their ability to detect trace concentrations of biologically molecules [8,57,58]. They have been employed in a variety of applications which include detecting single nucleotide mismatches in a single strands of DNA binding [59], as well as detecting proteins [60], small molecules, and even microorganisms [61]. This incredible sensitivity and versatility comes from the charge sensing capabilities of the semiconducting layer. Most biosensors of the type we are using work by means of a semiconducting layer with a functionalized insulator between it and the electrolytes in solution around it [58]. Their high sensitivity, comes from the charge sensing surface formed by the FET. In our case, the thin layer of polydopamine serves as both the functionalized layer and the FET, allowing us to detect changes in the local charge environment of the sensing electrode cause by the covalent modification of just a few catecholamine on the surface [24,25]. This is due to the fact that at neutral pH, SNOs are charged molecules, and such change the electrical properties of the FET (Fig 4B). A potential drawback to this is that since EISFETs rely on electrolytes as a charge carrier, they are sensitive to changes in pH of the solution (Fig 5) [62], making false positives due to fluctuations in pH and ionic concentrations of the running buffer a significant problem. This problem can be overcome by ensuring all biological samples are diluted at least 100-fold before they’re exposed to the sensor, and that the running buffer’s buffering capacity is capable of absorbing and changes in pH due to the sample being tested.

## Conclusions

We have developed a FET capacitive biosensor, which employs a polydopamine layer that acts as both the functional and semiconducting component of the sensor. This technology is the most sensitive method to date for selectively detecting small molecule SNOs in complex biological samples. It relies upon the specific interaction between polydopamine and S-nitrosothiols, allowing us to make a robust histochemical sensor for nitrosothiols, sidestepping the problem of producing reliable antibodies against SNOs, which would be required for functionalizing most existing FET capacitive sensors. These sensors will allow for the examination of the role of small molecule SNOs in breathing and blood pressure regulation, cystic fibrosis, asthma, pulmonary hypertension, and a host of other diseases and biological functions.

## Supporting information

S1 Appendix Additional methodology. This file contains additional text explaining the mathematics behind the normalized charge response as well as the exact method we used for signal processing.
(DOCX)
