# Supplementary material for: Detection of trace concentrations of S-nitrosothiols by means of a capacitive sensor
Source: PLoS One. 2017 Oct 26;12(10):e0187149. doi: 10.1371/journal.pone.0187149 (PMC5658150; doi:10.1371/journal.pone.0187149)
Supplement: S1 Appendix — This file contains additional text explaining the mathematics behind the normalized charge response as well as the exact method we used for signal processing. (DOCX) [file pone.0187149.s001.docx]

# Methods

## **Functionalizing Electrodes**

We cut the tip off of a 5µm carbon fiber microelectrode with a razor blade. We then placed it in a 1 MOhm head-stage, which was attached to an amplifier (Chem-Clamp, Dagan Corporation), and placed in plating buffer with a Ag-AgCl ground pellet (E206, Warner Instruments). The input voltage was set to 1:10 and a saw tooth potential was applied across the electrode by a ITC-1600 (HEKA Corporation) ramping between -6 V and 6.5 V every 24 s for the duration of the run. The Dagan amplifier reduces the signal by 10-fold such that the voltage applied across the tip of the electrode varies between -650 mV and 600 mV (**Fig. S1A**). We first run this saw tooth potential for 100 s in plain plating buffer with no dopamine to establish a baseline. We then perfuse the system with 50 ml of 1 mg/ml of dopamine in plating buffer and again applied the saw tooth potential for 100 s to ensure that polydopamine is indeed plating on the tip of the electrode by comparing the current output of the amplifier to the baseline run (**Fig. S1B**). If polydopamine is plating on the tip of the electrode, we then complete the process by running the same saw toothed potential for a further 900 s, and data are saved to document that the electrode was coated properly. Next, the electrode should be removed from plating buffer and places in running buffer for 30 minutes to finish functionalizing. Finally, the electrode should be removed from running buffer and dried if not used.

It is important to perform a short, 100 s run once dopamine is added to compare it to the baseline run to check that the electrode is coating properly before performing the 900 s plating run (**Fig. S1C**). Often, parylene coated carbon fiber electrodes such as those used in these experiments will have their parylene insulating coat seal over the exposed carbon on the tip of the electrode as it is cut, and this will prevent polydopamine from electroplating on the tip of the electrode during functionalization. It is also important that parylene coated electrodes spend the minimum amount of time they can in buffer as aqueous buffer slowly dissolves the parylene coating and renders the electrodes unusable. A capacitive biosensor will only work if the insulating coating on the electrode is intact as pinholes in the insulating layer abolish the signal.

## **Signal Processing**

The resulting recordings for the sensing electrodes were saved and the data were processed by first performing a Discrete Fourier Transform on it and notch filtering by zeroing every frequency that did not fit equation (1):

f = 2.5 Hz * (2 * i - 1) (1)

Where f is the frequency of the element in the Fourier transformed data and i is an integer between 1 and 10000. This was done because 2.5 Hz is the fundamental frequency which corresponds to 400 ms or the time it takes to perform a single cycle step potentials during a recording, and so all interesting changes in current must be contained in the 2.5 Hz sine wave, or one of the other odd numbered modes of the 2.5 Hz sine wave. The data were then converted back to the time domain, the 75 repetitions of the signal are averaged together, and the baseline recording is subtracted from the Blank Injection and the Blank Washout recordings. The Blank Washout Injection was subtracted from the Sample Injection and the Sample Washout recordings to prevent a false positive from machine drift, and the subtracted curves are recorded as raw output. We integrated under the first 4 ms of the subtracted curves to calculate the total charge accumulated in the initial charging phase of the sensing electrodes and take the absolute value. These four numbers represented the difference in charge accumulated on all capacitors in the circuit during the initial fast charging phase of the electrode response.

The total charge accumulation in the first 4 ms depends on many factors including electrode length, electrode width, ionic strength of the running buffer, and whether the polydopamine surface interacted with an analyte. To compare data between individual electrodes and individual runs, the data must be normalized such that the inter-electrode differences can be ignored. To do this we first normalized the charge accumulation:

n = (s – b) / ( s + 2 * b) (2)

Where n is the normalized response, s is the accumulated charged of the electrode after the sample washout, and b is the accumulated charge after the blank washout. This produces a value between -1 and 1 where negative values represent a signal drift in blank injection relative to the sample injection, or a negative signal, and positive numbers represent a signal change upon sample injection compared to blank injection. Since all cases where the baseline drift is higher than the sample injection are not interesting, we further simplify our normalized response:

r = (s – b + | s – b|) / (2 * s + 2 * b) (3)

Where r is the simplified response, and is equal to 0 is n is negative and equal to n if n is positive. In general, r ranged between 0 and 0.27 for a blank injection as the sample or a negative result, and 0.28 and 1 in the presence of a small molecule SNO.

## **Quality Controls**

When first functionalized, sensing electrodes should be tested by first injecting running buffer onto the sensor as a negative control to ensure that the electrodes are properly functionalized. Improperly functionalized electrodes will continue to drift in their accumulated charge giving a false positive. If this occurs, they should be allowed to incubate in running buffer for a longer period (at least 1 hour) before repeating the negative control. Once one to two successful negative controls have been run (one for well-functioning sensing electrodes and a second for sensing electrodes which have needed additional incubation time), a positive control should be run by injecting 100 µl of 10 fM SNO-Cysteine incubated in 10 mL of running buffer onto the sensing electrodes to discern a positive response. Some electrodes will fail their positive control because non-semiconducting dopamine aggregates form on the surface; these must be refunctionalized completely. In our experience, once a set of sensing electrodes has passed these quality control measures, they can be used for 1 to 5 sensing experiments before they will need to be refunctionalized and retested.

# Results

## Raw Experimental Data

The raw current versus time curves produced during a single recording reflect the charging of a circuit containing multiple capacitors. These capacitors represent a Helmholtz interaction along the surface of the electrode between the surface of the electrode and the electrolytes in the buffer solution. Negatively charged chloride ions are absorbed by the Ag-AgCl pellet, leaving behind positively charged sodium ions. This charge is neutralized by an electrical current generated by the pre-amplifier. This represents many capacitors in parallel with the smallest of them representing the capacitor formed by the polydopamine coated tip of the sensing electrode. This capacitor determines the initial fast rise of current measured by the pre-Amplifier upon voltage change. To state this another way, the fast rise in the resulting charging curve contains all of the data regarding whether or not a sensor has interacted with an analyte. This can be seen by focusing on the first four milliseconds after the voltage across the sensing electrode is stepped from 0 mV to +50 mV. Individual traces can be contaminated with electrical noise, so filtering the resulting data curves is recommended in order to prevent false positives or negatives due to a noise source making either the Blank Washout or the Sample Washout recording appear to shift away from the sensor’s equilibrium charging current.

## **Filtering Raw Data**

We took the Fourier Transform of our current recordings and examined the resulting frequency spectrum (**Fig. S2A and S2B**). The vast majority of the information in these frequency spectrum is contained within frequencies, which satisfy equation 1. These frequencies represent common responses in the time domain to stepping between 0 mV and 50 mV and back to 0 mV. As any real change in capacitance due to analyte interaction will result in a change in the charging profile of all 75 charging curves collected during a single recording, we can directly infer that any signal we wish to extract from these data will be contained in the frequencies which satisfy equation 1. We notch filtered our data, setting all frequencies which do not satisfy equation 1 to 0 (**Fig. S2C**). We then performed an inverse Fourier Transform to return to the time domain with the majority of the noise filtered out of our original curves. While there was still an obvious shift in the sample curve upon exposure to small molecule SNOs, the charging currents were still quite similar. For this reason, we calculated the difference between the Baseline and Blank Washout Curve, and the Blank Washout and Sample Injection Curves to be better able visually to inspect the raw data (**Fig. S2D**). As expected, the difference between these curves was largest during the first few milliseconds after the voltage step changes.

## **Normalized Charge Accumulation**

We integrated under the first 4 ms of the subtracted curves. This represents the amount of charge, which accumulates on the tip of the sensing electrode directly after a change in voltage across the circuit. The charge accumulated in this time is strongly dependent on the length and precise diameter of a given carbon electrode, and any change in the rate at which it accumulates charge is also proportional to the basal level of charge accumulation. In order to compare signals of various electrodes, we normalized the accumulated charge using equation 3. In our experience, adequate electrodes should produce a normalized charge response of 0.012 ± 0.008 which is produced by fluctuation in pH and electrical drift inherent in the pre-amplifiers. As an analyte interacts with the tip of the sensing electrode, it changes the amplitude and kinetics of the fast charging current, shifting the value of the normalized charge accumulation. We constrained our curve subtraction to ensure that the difference is always positive, making the normalized charge accumulation appear to rise. This response ratio generally gave an average response of 0.65 ± 0.10 upon detection of a small molecule SNO.

## **Sensor Saturation**

The polydopamine coated tip of the sensing electrode represents a finite surface area for interaction with SNOs in solution, and as such, newly functionalized electrodes will always lose sensitivity to SNOs over time. This is best be seen by functionalizing an electrode and exposing it increasing concentrations of GSNO (**Figure S4**). The raw current response to incubation with GSNO at first increases with concentration as the higher concentration coats a greater proportion of the electrode’s surface during incubation. As we repeatedly exposed it to increasing GSNO concentrations, the response that each electrode gave was first blunted and finally abolished after 4 experiments. This represents the polydopamine surface of the sensing electrode tip reacting with GSNO in solution and preventing further interaction. The precise rate at which electrodes lose sensitivity depends on the SNO concentrations they are exposed to. At sub-fM concentrations a given set of electrodes can be exposed several times before they become unusable. As the SNO concentration increases, the proportion of the tip, which is rendered unusable in a single experiment increases, until at high pM concentrations a single electrode will be rendered useless after a single experiment. For this reason, we recommend preparing freshly functionalized and tested sensing electrodes for each new biological sample.

# Figures

**
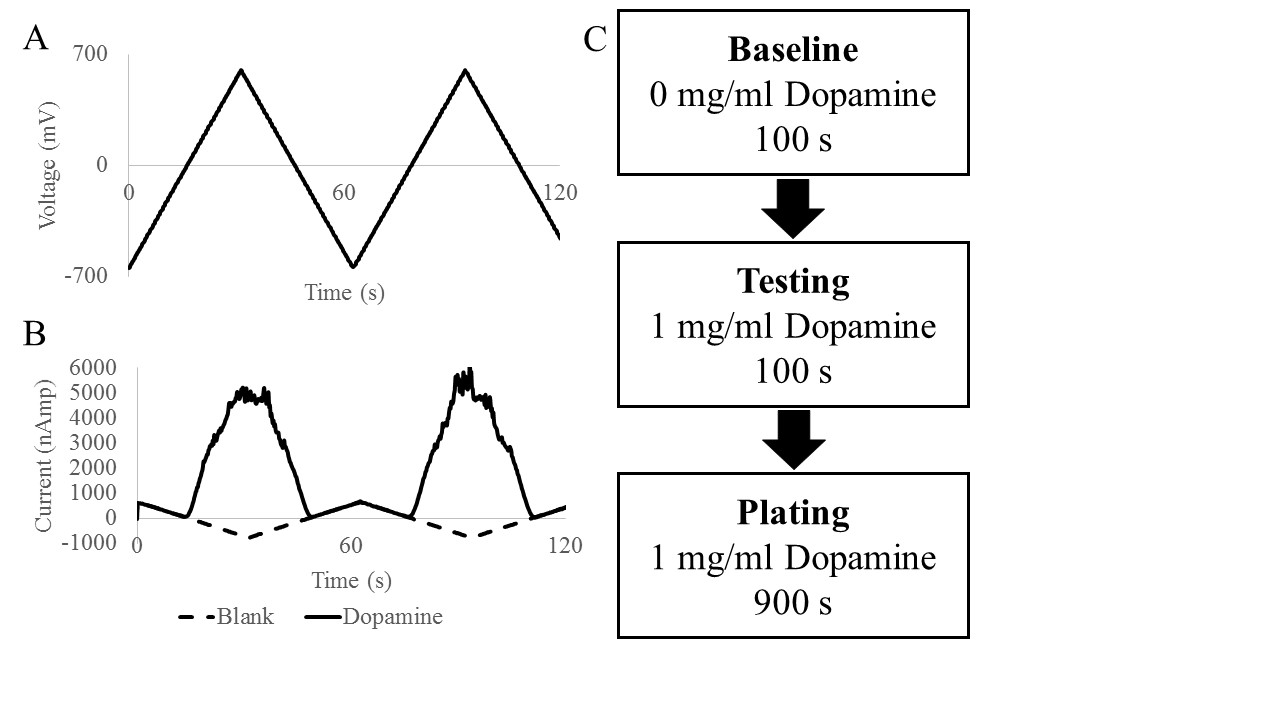
**

**Fig. S1:** An example current trace of the input voltage (A) and amplified output current (B) of a freshly cut parylene coated carbon fiber electrode sitting in a bath of either plain buffer (dotted line) or 1 mg/ml dopamine hydrochloride (solid line). The rise in current is proof that dopamine is polymerizing on the surface of the carbon electrode and this data should be collected during each and every functionalization step to ensure proof that the carbon electrode was well coated with polydopamine prior to fixation in formaldehyde.

**
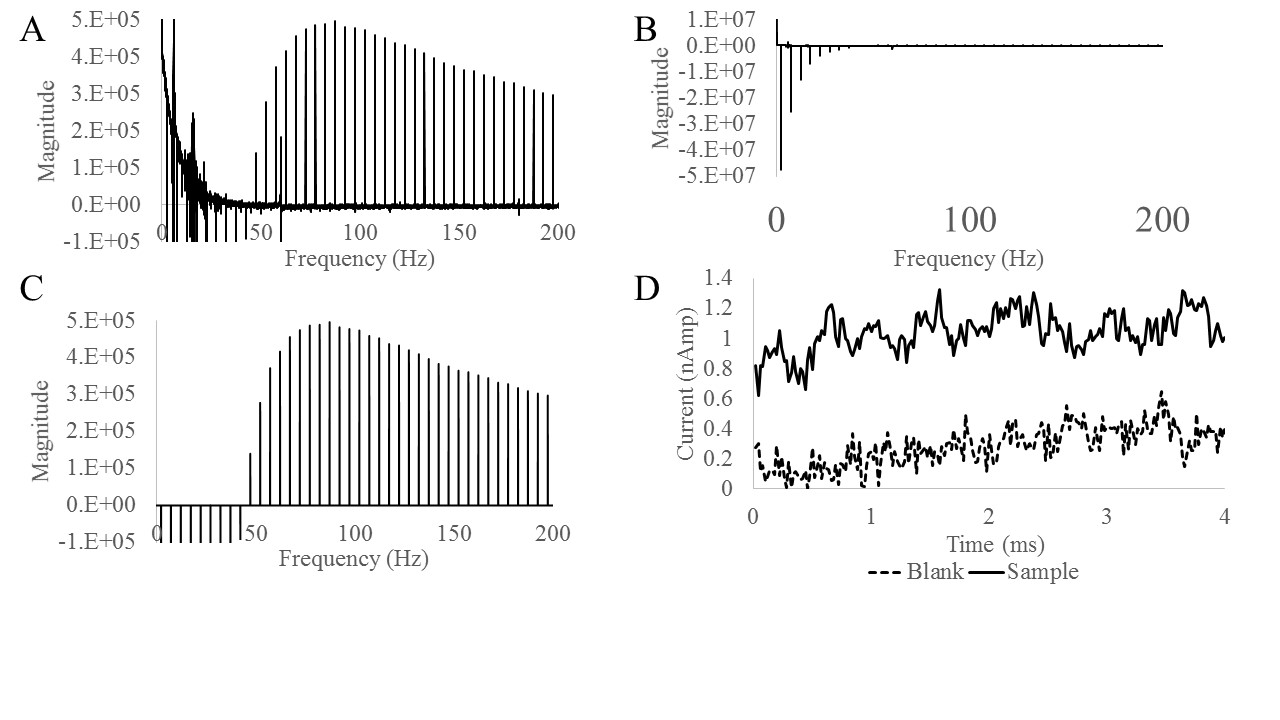
**

**Fig. S2:** A zoomed in (A) and zoomed out (B) example trace of the Fourier transform of a single run. The large, regular spikes in the curve represent frequencies which are odd modes of the 2.5 Hz frequency corresponding to the time it takes our system to complete a single voltage step cycle. Data was notch filtered (C) to exclude all information, which does not have a periodicity in step with our experiment to exclude random electrical noise in our signal before the data was converted back to the time-domain and the baseline data was subtracted (D). The fast current rise during the first 4 ms changes dramatically upon the addition of a SNO, when compared to a blank running buffer injection.

**
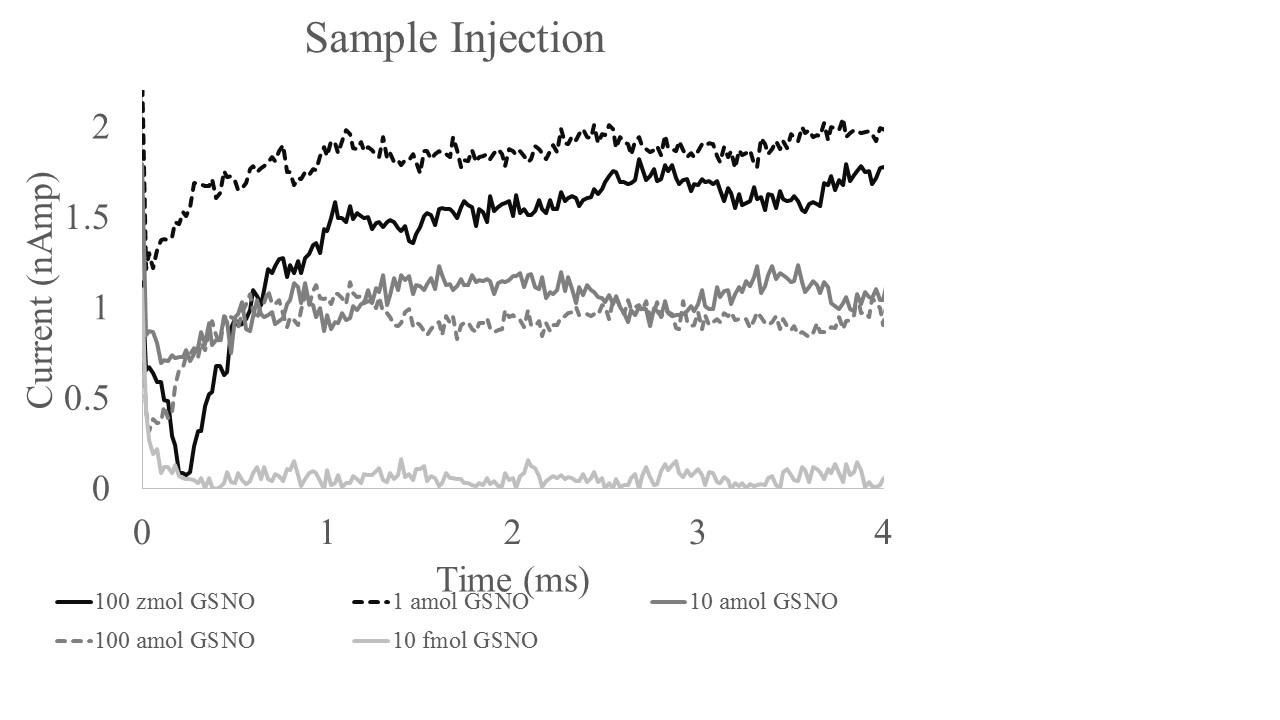
**

**Fig. S3:** The baseline subtracted signal of a freshly functionalized carbon-fiber electrode as it was sequentially exposed to 100 zmol, 1 amol, 10 amol, 100 amol, and finally 10 fmol of GSNO. The electrode was washed and allowed to stabilized after each run, and a fresh well was provided each time such that the starting concentration of GSNO in every experiment was 0. There was an initial increase in the capacitive charging current upon exposing it to 100 zmols and then 1 amol GSNO. However, the response soon declined, until the sensing electrode no longer showed a difference in charging current upon exposure to 10 fmol of GSNO. This shows how sensing electrodes lose their ability to sense as the free quinone groups on the polydopamine surface become covalently bonded to thiols, blocking them from future interaction.
